# Understanding the determinants of self-reported asthma in Turkey: insights from national data on individual, lifestyle, socioeconomic, and healthcare access factors

**DOI:** 10.1186/s12890-024-03159-7

**Published:** 2024-07-18

**Authors:** Aslı Davas, Meltem Çiçeklioğlu

**Affiliations:** Department of Public Health, Ege University Faculty of Medicine, Bornova, Izmir, Türkiye 35100

**Keywords:** Asthma, Socioeconomic determinants, Healthcare access, Unmet need, Lifestyle factors, Inequity

## Abstract

Asthma, influenced by genetic, environmental, and social factors is leading to poor outcomes and preventable mortality due to inadequate care and limited access to effective treatments. This study aimed to analyze self-reported asthma prevalence in Turkey, focusing on its determinants, such as individual factors, lifestyle, socioeconomic status, and healthcare access.

This study conducts a secondary analysis of the 2019 Turkiye Health Survey (THS), employing a nationally representative cross-sectional design by the Turkish Statistical Institute. The sampling utilized a stratified, two-stage cluster sampling method, with data from 16,976 adults (aged 15 years and older) analyzed for asthma determinants. The independent variables are categorized into four domains: individual factors, lifestyle assessment, socioeconomic factors, and access to the healthcare services.

The prevalence of asthma is 9.8%, varying significantly across demographics. Higher asthma rates are observed among older, divorced/widowed individuals, those with communication difficulties, and obese individuals. Cost-related unmet healthcare needs and appointment scheduling delays increase asthma risk. Logistic regression models identified age, marital status, obesity, education level, and healthcare access as significant predictors of asthma.

This study underscores the multifaceted determinants of asthma in Turkey, highlighting the necessity for targeted interventions addressing individual, lifestyle, socioeconomic, and healthcare access factors.

## Introduction

Asthma is a widespread chronic respiratory condition that significantly impacts global health, affecting a substantial portion of the population, ranging from 1% to 29% across various countries [1]. This disease imposes a considerable burden, leading to premature mortality and reduced quality of life. It contributes significantly to disability-adjusted life years (DALYs) reduction, accounting for approximately one-fifth of all losses related to chronic respiratory diseases and ranking 34th in global disease burden assessments [2].

In different regions of Turkey, adult asthma studies, mainly using the European Community Respiratory Health Survey (ECRHS), reported asthma prevalence ranging from 1.2% to 9.4% and asthma-like symptom prevalence between 9.8% and 27.3%. The multicenter PARFAIT study by the Thoracic Society revealed a nationwide asthma prevalance of 7.1% in males and 9.0% in females [3, 4].

Despite the lack of standardized definitions of asthma, diagnostic criteria (such as spirometry) that can be used in all countries, and the lack of consensus between surveillance programs and questionnaires that have led to imprecise estimates of the incidence, prevalence, and burden of the disease, there is strong evidence that the incidence of asthma has increased significantly over the past 50 years. Despite the global political commitment, progress in reducing the burden of asthma has been insufficient, particularly in low-income countries [5].

Asthma is a complex condition influenced by genetic, environmental, and social factors. Social determinants of health (SDHs) encompass various aspects, such as early childhood development, education, gender, working conditions, income, social status, environment, lifestyle, and access to healthcare [6]. Disruption in these areas leads to health disparities, especially in respiratory diseases, compared to others [7]. Studies highlight low socioeconomic status (SES) and poverty-related factors as strongly linked to asthma development and poor outcomes, such as lung function decline and exacerbations. Preventable asthma mortality and severe attacks often result from inadequate care and limited access to effective treatments due to a lack of government support and policies [5, 7, 8].

Understanding asthma's inequalities requires a multifaceted approach, considering a range of risk factors across different levels. The World Health Organization's conceptual framework on Social Determinants of Health illustrates how socioeconomic and political contexts (e.g., government, policies, and cultures) influence structural determinants (e.g., social class, education, income) and subsequently impact individual behaviors (e.g., obesity, smoking) and healthcare access [8]. While some studies in Turkey have focused on asthma-related factors, national research linking asthma to lifestyle, social determinants, and healthcare access is lacking [3]. Addressing these gaps is essential for developing effective strategies to mitigate the impact of asthma and improve public health outcomes in Turkey.

This study aimed to analyze the prevalence of self-reported asthma in Turkey and its determinants, such as individual factors, lifestyle, socioeconomic status, and healthcare access. It also addresses gaps in national research by investigating the link between asthma and lifestyle choices, social determinants, and healthcare accessibility in the country.

## Methods

### Study design and setting

The Turkish Statistical Institute (TurkStat) conducted the 2019 Turkiye Health Survey (THS), a cross-sectional study, between September and December 2019. For national representation, the sampling of surveys utilizes a stratified, two-stage cluster sampling method based on the "Address-Based Registry System". The THS 2019 included 17,084 adult individuals (aged 15 years and older) across Turkiye. The study methodology excluded individuals residing in dormitories, prisons, hospitals, nursing homes, small villages or hamlets.

The microdata set of the 2019 Health Survey was officially requested and obtained from TurkStat to develop a model to explore the determinants of asthma in Turkey [9, 10]. After the data cleaning process, we included 16,976 individuals aged 15 years and older in our analyses.

The dependent variable of this study was any positive answer to the question “During the past 12 months, have you had asthma (allergic asthma included)?”

The study employed a four-domain approach to analyze independent variables influencing asthma prevalance. Individual factors were evaluated by age, marital status, and difficulties in Turkish communication. Lifestyle assessment was used to assess the presence of obesity (measured via body mass index), and activity levels were categorized as inactive, minimally active, and highly active using MET calculations and smoking. Socioeconomic factors included education, employment status, and access to public health insurance. Healthcare service accessibility is assessed by cost-related unmet healthcare needs, delays experienced in accessing healthcare within the past 12 months due to lengthy appointment scheduling, and delays encountered in healthcare access within the past year due to transportation or distance constraints.

Detailed descriptions of each independent variable can be found on the metadata of the Turkiye Health Survey page of the Turkish Statistical Institute [9, 10]. Additionally, an appendix table titled "The Survey Questions and Categorization of Related Variables" has been added for easier reference.

### Ethical approval

The principles of the Declaration of Helsinki were followed, meaning that the survey results were published at an aggregate level and that the anonymity of the interviewed individuals and households was fully secured. Before interpreting the results, the researchers obtained the consent of the Ethical Board from the School of Medicine at Ege University.

### Statistical analyses

We evaluated the associations between independent variables and asthma using chi-square analysis. The data are presented as numbers and percentages. Variables that were significant according to Pearson's chi-square tests were considered independent variables in multivariate logistic regression models. We employed four logistic regression models in the analyses and calculated odds ratios (ORs) and 95% confidence intervals (CIs). Hosmer and Lemeshow's test yielded a probe value of more than 0.05 for all four models. For all analyses, the p value was considered to be < 0.05. We performed the analysis using the Statistical Package for Social Sciences (SPSS) version 26.

## Results

The participants in the study group had an average age of 44.0±17.7 years, 33.9% of whom were younger than 35 years. The percentage of women was 54.6%, and 68.6% of the respondents were married.

A minority of the group (3.1%) reported challenges in native language communication. Furthermore, 15.0% were unemployed, while 32.8% identified as homemakers. Notably, 15.4% indicated experiencing cost-related unmet healthcare needs (Table 1). The overall asthma prevalance was 9.8%. However, the prevalence of asthma varies significantly across individuals, lifestyles, and socioeconomic factors. Specifically, we observed higher asthma rates among women (12.7%), divorced or widowed individuals (19.3%), and those facing communication difficulties in Turkey (21.6%). Moreover, obese individuals (21.5%), inactive individuals (11.6%), those with less than 5 years of education (14.9%), and those covered by public health insurance (10.0%) exhibited elevated asthma prevalence. Moreover, asthma prevalence was notably greater among homemakers (14.3%), individuals with the lowest income (13.0%), and those with high levels of cost-related unmet needs (14.9%). Notably, delays due to lengthy appointment scheduling (11.9%) and delays due to distance or transportation constraints (14.9%) were statistically significant (Table 1).

**Table 1:** Descriptions of the independent variables and their associations with asthma (15+ years, Turkiye, 2019, *n*=16976)

			Asthma +		Asthma -		
Variables	Total N (16976)	%^a^	N (1658)	R%^b^	N (15318)	R%^b^	*p*-value
Sex							0,0001
Men	7711	45,4	484	6,3	7227	93,7	
Women	9264	54,6	1174	12,7	8090	87,3	
Age							0,0001
<35	5762	33,9	257	4,5	5505	95,5	
35-44	3362	19,8	223	6,6	3139	93,4	
45-54	2896	17,1	336	11,6	2560	88,4	
55-64	2500	14,7	373	14,9	2127	85,1	
>65	2456	14,5	469	19,1	1987	80,9	
Marital status							0,0001
Never married	3591	21,2	145	4,0	3446	96	
Married	11642	68,6	1176	10,1	10466	89,9	
Divorced/widowed	1743	10,3	337	19,3	1406	80,7	
Any difficulty in native language communication							,00001
No	16449	96,9	1547	9,4	14902	90,6	
Yes	527	3,1	111	21,1	416	78,9	
BMI *n*=16850	N (16850)		N (1637)		N (15213)		0,0001
Normal	13082	77,6	1033	7,9	12049	92,1	
Overweight	2775	16,5	391	14,1	2384	85,9	
Obese/Morbid obese	993	5,9	213	21,5	780	78,5	
Physical Activity							
Inactive	10591	62,4	1229	11,6	9362	88,4	
Minimal active	5906	34,8	411	7,0	5495	93,0	
Very active	479	2,8	18	3,8	461	96,2	
Smoking							0,0001
Regular and occasional	5191	30,6	375	7,2	4816	92,8	
Never	9206	54,2	958	10,4	8248	89,6	
Quit	2579	15,2	325	12,6	2254	87,4	
Education							0,0001
<5 years	7775	45,8	1161	14,9	6614	85,1	
5-8 years	2951	17,4	167	5,7	2784	94,3	
>9 years	6250	36,8	330	5,3	5920	94,7	
Have universal health insurance							0,0001
Yes	15636	92,1	1571	10,0	14065	90	
No	1340	7,9	87	6,5	1253	93,5	
Employment							0,0001
Employed	6423	37,8	373	5,8	6050	94,2	
Unemployed	2553	15,0	168	6,6	2385	93,4	
Housemaker	5562	32,8	797	14,3	4765	85,7	
Retired	2438	14,4	320	13,1	2118	86,9	
Household income							0,0001
Lowest	5248	30,9	680	13,0	4568	87	
Low	6614	39,0	597	9,0	6017	91	
Middle	3229	19,0	260	8,1	2969	91,9	
Upper	1885	11,1	121	6,4	1764	93,6	
Cost-related unmet need (*n*=16417)	N (16417)		N (1646)		N (14771)		0,0001
No	13894	84,6	1269	9,1	12625	90,9	
Yes	2523	15,4	377	14,9	2146	85,1	
Delay due to lengthy appointment scheduling (previous year)(*n*=16394)	N (16394)		N (1645)		N (14749)		0,0001
Yes	4176	25,5	497	11,9	3679	88,1	
No	12218	74,5	1148	9,4	11070	90,6	
Delay due to distance or transportation constraints (previous year)	N (16438)		N (1645)		N (14793)		0,0001
Yes	1902	11,6	284	14,9	1618	85,1	
No	14536	88,4	1361	9,4	13175	90,6	

^a^Column percentage

^b^Row percentage

Four multivariate models were constructed to assess the influence of individual characteristics, lifestyle factors, socioeconomic determinants, and access to the healthcare system. As new variables were added to each model, Nagelkerke's R-squared values increased.

In the first model assessing individual characteristics, it was found that older age increased the risk of asthma, and among the divorced/widowed individuals, there was a 1.6-fold (95% CI: 1.3-2.1) increase in risk, while those experiencing difficulty in communication in Turkish showed a 1.6-fold (95% CI: 1.3-2.0) increase in risk(Table 2).

**Table 2:** Multivariate analyses of determinants of asthma (15+ years, Turkiye, 2019)

Variables	Model 1 Individual characteristics OR (95%CI)	Model 2 + Lifestyle OR (95%CI)	Model3 + SES OR (95%CI)	Model 4 + Access to healthcare system OR (95%CI)
Women (Ref: Men)	2,1(1,9-2,4)	2,1(1,8-2,4)	2,0(1,7-2,3)	1,9(1,6-2,2)
Age groups (Ref. <35)				
35-44	1,2(1,1-1,4)	1,3(1,1-1,5)	NS	1,2(1,0-1,4)*
45-54	1,6(1,4-1,9)	1,6(1,4-1,9)	1,4(1,2-1,7)	1,5(1,3-1,8)
55-64	3,0(2,5-3,6)	2,7(2,2-3,2)	2,2(1,8-2,7)	2,4(1,9-2,9)
>65	3,9(3,2-4,8)	3,3(2,7-4,1)	2,5(2,0-3,2)	2,8(2,2-3,4)
Marital status (Ref: Never married)				
Married	1,2(1,0-1,4)*	1,2(1,0-1,4)*	NS	NS
Divorced/widowed	1,6(1,3-2,1)	1,5(1,2-1,9)	1,5(1,2-2,0)	1,5(1,1-1,9)
Any difficulty in native language communication (Ref: No)	1,6(1,3-2,0)	1,5(1,2-1,9)	1,4(1,1-1,8)	1,3(1,0-1,7)*
Obesity (Ref: Normal weight)				
Overweight		1,4(1,1-1,7)	1,4(1,1-1,6)	1,3(1,1-1,6)
Obese+Morbid obese		1,9(1,6-2,3)	1,8(1,5-2,2)	1,8(1,5-2,1)
Activity (Ref: Very active)				
Minimal active		1,3(1,1-1,4)	NS	NS
Inactive		1,7(1,0-2,7)*	1,2(1,1-1,4)	1,3 (1,1-1,4)
Smoking (Ref: Regular and occasional smokers)				
Never		1,3(1,2-1,6)	1,3(1,1-1,6)	1,4(1,2-1,6)
Quitted		1,4(1,2-1,7)	1,5(1,3-1,7)	1,5(1,2-1,8)
Education (Ref: >9 years)				
<5 years			1,5(1,3-1,8)	1,5(1,3-1,8)
5-8 years			1,4(1,2-1,7)	1,4(1,2-1,7)
Have universal health insurance (Ref: no insurance)			NS	NS
Income (Ref: High)				
Lowest			1,2(1,1-1,3)	NS
Low			NS	NS
Moderate			NS	NS
Employment (Ref: retired)				
Employed			0,7(0,6-0,9)	0,7(0,6-0,9)
Unemployed			NS	NS
Homemaker			NS	NS
Cost-related unmet need (previous year, Ref: No)				1,6(1,4-1,8)
Delay due to lengthy appointment scheduling (previous year, Ref: No)				1,3(1,1-1,4)
Cox & Snell R2	0,044	0,050	0,054	0,058
Nagelkerke R2	0,094	0,106	0,115	0,121

^*^P<0,05

In Model 2, which was developed by adding lifestyle factors, there was no change in the impact of individual characteristics; additionally, both obesity and activity were observed to increase the risk of asthma.

Model 3, which included socioeconomic variables, showed that the effects of individual characteristics and obesity persisted, but contrary to univariate analyses, only low education levels and the lowest income group increased the risk of asthma. The risk of asthma disappeared for minimal activities in Model 3 (Table 2).

The addition of variables related to access to healthcare services in Model 4 did not alter the effects of the variables in the previous three models except for the effect of lowest income. The risk of asthma increased by 1.6-fold (95% CI: 1.4-1.8) due to cost-related unmet healthcare needs and by 1.2-fold (95% CI: 1.1-1.4) among those experiencing delays due to lengthy appointment scheduling. A slight decrease in the impact of gender was observed after adding socioeconomic and access to healthcare variables. A similar effect was observed for difficulty in communication in Turkish(Table 2).

## Discussion

This study pioneers the investigation of the combined influence of individual characteristics, lifestyle factors, socioeconomic determinants, and healthcare accessibility on asthma within a population that is representative of Turkey. Studies on asthma or its prevalence in Turkey are primarily hospital-based, resulting in a wide range of prevalence rates [3, 4, 11–15].Our study relies on self-reported asthma and is not based on a specific phenotype; we did not encounter a similar study representing both rural and urban areas across Turkey. Nevertheless, our findings align with both population-based studies conducted in specific regions of Turkey and are even more robust than those of other studies [4, 16–18]and within the range of global prevalence estimates of ever-existing asthma derived from 220 population-based studies [16]. The multivariate models used in our analysis allowed us to assess the independent contributions of various factors to asthma risk while controlling for potential confounders. Interestingly, we observed that the impacts of individual characteristics, lifestyle factors, and socioeconomic factors remained consistent across the different models, highlighting their robust associations with asthma outcomes.

Individual factors are among the most frequently studied determinants affecting asthma prevalance in the literature. In our study, both univariate and multivariate analyses consistently showed a greater prevalance of asthma among women, older individuals, specifically divorced or widowed individuals, and those experiencing difficulties in communication in Turkey in all four models.

In this study, the prevalence of asthma in Turkey increased with age, particularly among individuals aged 45 years and older. Global research as well as some national research indicates a rise in adult asthma prevalance across age groups, notably from young adults through middle age [11–13]. The SNAPSHOT study, which was conducted in five Middle Eastern countries, including Turkey, and two population-based studies, which were conducted in two of the provinces of Turkey, support this trend of increasing asthma prevalance with age, as observed in our study [15, 17]. This outcome could be explained by the degenerative alterations in the respiratory system associated with aging, as well as the coexistence of chronic pulmonary conditions in the elderly population. Physiologic and immunologic changes in aging populations complicate asthma diagnosis and management. Factors such as nonadherence, tobacco use, inhaler difficulties, and corticosteroid-related side effects contribute to decreased disease control across age groups [18, 19].

In accordance with national and international research, our study revealed a higher rate of asthma in women [4, 13, 15–17, 20, 21]. There are varying results from different countries regarding the impact of marital status on asthma prevalance. While one study found no association between relationship status and asthma [22], a study from India reported a greater prevalance of asthma among divorced or widowed individuals [23]. In our study, the elevated asthma rates among divorced or widowed individuals may be attributed to factors such as a greater proportion of divorced/widowed women of older age and lower income levels. Furthermore, stress, either independently or leading to unhealthy eating habits, could also contribute to the increased frequency of asthma in our study.

We observed a greater prevalence of asthma among individuals facing difficulties in native language communication. This finding suggests a potential causal relationship between language barriers and asthma prevalence. Notably, significant racial and ethnic disparities regarding asthma morbidity, prevalence, mortality rates, and responses to medications have been reported in the literature [20, 24], which aligns with the findings of our study. However, it is important to note that Turkey's census categories do not explicitly define racial and ethnic groups. Despite Turkish being the primary language of instruction in educational institutions, a considerable portion of the population speaks Kurdish (14%) and Arabic (2%), with the rest belonging to other language groups [21]. A study conducted in Turkey shed light on the challenges encountered by individuals lacking proficiency in the official language when accessing healthcare. These challenges include limited access to health information, strained patient-provider relationships, delays in seeking medical care, reliance on others for healthcare access, low adherence to treatment regimens, dissatisfaction with healthcare services, and difficulties exercising healthcare right [25].

Although a relationship between both obesity and physical activity with asthma was found in the univariate analysis, minimal physical activity was not found to be associated with asthma in Models 3 and 4. The literature has shown that obesity is associated not only with the development of asthma but also with asthma-related limitations in activities such as sports, indicating its role in the manifestation of asthma [26–28]. Given the increasing trend of obesity in Turkey [29, 30], it is crucial to enhance preventive and therapeutic services related to obesity in healthcare [30, 31] Following the inclusion of socioeconomic and healthcare access variables in the model, the removal of minimal physical activity as a risk factor suggests that even the addition of walking paths in socioeconomically disadvantaged areas could be beneficial for the control of asthma [31].

Our study findings indicate that the prevalence of asthma is highest among former smokers, with nonsmokers showing a greater frequency of asthma than regular or occasional smokers. This observation raises several important questions. First, it underscores the complex relationship between smoking status and asthma development, highlighting the need for a nuanced understanding of these associations [32]. Second, smoking cessation may not immediately alleviate the risk of asthma, as evidenced by the elevated asthma rates among former smokers [33, 34]. These complexities underscore the necessity for further research to elucidate the mechanisms underlying the observed patterns and to inform targeted interventions for asthma prevention and management. The cross-sectional nature of our study adds a layer of complexity to accurately interpreting cause–effect relationships. Similarly, a previous cross-sectional study in Turkey indicated a lower smoking prevalence among people with asthma than among the general population, potentially contributing to these findings [34]. Furthermore, factors beyond smoking, especially in allergic and elderly populations, play a role in the complex dynamics of asthma development. The prevalence of asthma associated with smoking seems to mirror that of the general population, yet asthma management tends to be more challenging and severe in active smokers [33]. These factors highlight the need for personalized approaches to asthma management, taking into account individual risk factors and lifestyle choices. However, further research is needed to better understand the relationship between smoking and asthma development in different populations.

In the literature, socioeconomic status has often been assessed either independently as education or in some studies as a combination of education and income. In this research, the effects of four different SES variables were evaluated both individually and collectively.

According to the univariate analyses, all socioeconomic status (SES) variables, such as education, insurance coverage, income, and employment, were found to be associated with asthma. However, in the multiple regression analyses, among these SES variables, the lowest income level group and lower education were identified as risk factors for asthma in Turkey, while employment appeared to act as a protective factor.

Education has consistently been found to be significantly associated with asthma in both univariate and multivariate analyses, consistent with the findings of numerous national and international studies [23, 24, 35, 36]. Education is not only crucial for asthma onset but also plays a significant role in asthma management [35, 37]. Its stability over time, unlike occupation and income, can vary throughout life, which can explain its continued impact across all models, particularly due to its influence on health literacy, which is a key determinant of treatment adherence.

While conflicting results due to confounding factors exist, an expanding body of research indicates a link between low income and increased asthma prevalence, and exacerbations [35, 36, 38]. In our study, although the lowest income level was significant in Model 3, this association disappeared when healthcare access variables were considered.

Additionally, the absence of a discernible impact of having health insurance in the multivariate analyses could be attributed to several factors. One possible explanation is the comprehensive coverage provided by universal health insurance in Turkey, which may have minimized the differences between insured and uninsured individuals regarding access to healthcare services related to asthma. These findings suggest that healthcare access could serve as a mediator between insurance, income, and asthma outcomes, highlighting the intricate interplay of socioeconomic factors in health outcome [8, 36].

In our study, we observed a significant disparity in access to healthcare services for asthma patients, particularly concerning cost-related issues and appointment unavailability. This finding is crucial for understanding the challenges in controlling asthma as a disease. Asthma imposes substantial financial burdens due to the high costs associated with diagnosis, treatment, and ongoing care, including expensive medications and specialized services [39, 40]. Research indicates that individuals with uncontrolled asthma experience higher rates of hospitalization and medication prescriptions, leading to increased healthcare costs for asthma-related and general healthcare needs. These disparities are observed globally, even in countries with universally funded healthcare systems [41–43].

Despite Turkey's relatively high health insurance coverage, access barriers related to cost persist. Analysis of global health expenditure data reveals a decline in Turkey's healthcare spending as a percentage of GDP, leading to heightened private healthcare expenses, mainly driven by out-of-pocket payments and private health insurance. This trend intensifies the financial burden on individuals seeking healthcare services [42]. Furthermore, the rapid expansion of healthcare privatization in recent years has resulted in a considerable migration of specialized services from the public to private sectors in Turkey, with poor patient satisfaction outcomes in the public sector [43]. Therefore, addressing healthcare access for asthma patients, starting from preventive services, is crucial and should be carefully addressed in healthcare policies.

This study was conducted not based on a specific asthma survey but rather by utilizing a single question related to asthma from a study aiming to identify health issues in Turkey. Limitations of this study include its reliance on self-reported asthma rather than clinical diagnosis, which may introduce recall bias and misclassification. Additionally, the study's cross-sectional design limits the ability to establish causal relationships between variables. The use of self-reported data also raises concerns about the accuracy and consistency of responses, particularly regarding sensitive topics such as smoking and income. Furthermore, the study did not differentiate between asthma phenotypes or severity levels, which could have provided more nuanced insights into the relationship between individual characteristics and asthma outcomes. There might be other unmeasured confounding factors that were not accounted for in the study, such as environmental and occupational factors, genetic predisposition, and exposure to allergens.

This study highlights the intricate effects of individual characteristics, lifestyle factors, socioeconomic factors, and healthcare accessibility on asthma prevalence in Turkey. Turkish women, older individuals, particularly divorced or widowed people, and those with communication challenges showed a greater prevalence of asthma. Tailored interventions focusing on behavioral changes and enhanced primary care services are crucial, especially for elderly people with asthma. Overall, our findings underscore the need for targeted interventions addressing language barriers in healthcare to improve asthma management and healthcare outcomes among linguistically diverse populations. The efficacy of intensive pulmonary rehabilitation programs, incorporating exercise training, dietary adjustments, and psychological support, has been demonstrated in improving the management of obese individuals with poorly controlled asthma, underscoring the value of such interventions in enhancing asthma control, physical health, and exercise performance.

Although initially related to asthma, minimal physical activity was not a significant risk factor in later models, suggesting that even basic exercise interventions can benefit asthma control, especially in disadvantaged areas. Smoking patterns underscore the need for personalized approaches and further research into smoking's impact on asthma. Education consistently influenced asthma prevalence, while low income was identified as a risk factor in Turkey. Employment acted protectively. The limited impact of health insurance suggests the importance of addressing healthcare access barriers through policy interventions. Despite high health insurance coverage, access barriers related to cost and appointment unavailability persist. Robust policies targeting preventive services and equitable access to specialized care are crucial, given the decline in healthcare spending and the growing private healthcare sector.

In conclusion, a comprehensive approach involving individualized interventions, lifestyle modifications, targeted healthcare policies addressing socioeconomic barriers, and equitable.

## Data Availability

This study is not a clinical trial. The data that support the findings of this study are available from the Turkish Statistical Institute, but restrictions apply to the availability of these data, which were used under license for the current study and are not publicly available. However, the data are available from the authors upon reasonable request and with the permission of the Turkish Statistical Institute. Contact person for the study’s data request: aslidavas@gmail.com The website where the data can be purchased: https://data.tuik.gov.tr/Kategori/GetKategori?p=Health-and-Social-Protection--101

## Appendix

**Table 3:** The Survey Questions and Categorization of Related Variables

**Questions**	**Variable**
**Individual Factors**	
What is your age?	Age
What is your gender?	Gender
What is your marital status?	Categorized as single, married, divorced, or widowed
Do you have difficulty in communicating and understanding with your mother tongue?	Categorized as no difficulty, any difficulty (some + a lot of)
**Lifestyle Assessment**	
How much do you weigh without clothes and shoes? How tall are you without shoes?	Obesity - BMI calculated and categorized as normal, overweight, obese/morbid obese
In a typical week, on how many days do you walk for at least 10 minutes continuously to get to and from places? How much time do you spend walking in order to get to and from places on a typical day? In a typical week, on how many days do you bicycle for at least 10 minutes continuously to get to and from places? How much time do you spend bicycling in order to get to and from places on a typical day? In a typical week, on how many days do you carry out sports, fitness, or recreational (leisure) activities for at least 10 minutes continuously? How many hours in total do you spend on sports, fitness, or recreational (leisure) physical activities in a typical week? How many minutes in total do you spend on sports, fitness, or recreational (leisure) physical activities in a typical week? In a typical week, how many days do you carry out activities specially designed to strengthen your muscles such as resistance training or strength exercises? In a typical day, how much time do you spend on sitting and resting?	Physical Activity - MET calculated and categorized as inactive, minimally active, very active
Do you smoke?	Smoking - Categorized as regular and occasional; never; quit
**Socioeconomic Factors**	
What is the highest education level you have completed?	Education - Categorized as <5 years (illiterate included); 5-8 years; >9 years
Are your treatment costs met by SSI?	Have Universal Health Insurance - Categorized as yes and no
What is your working status?	Employment - Categorized as employed (wage, salary or casual employee, employer/self-employed, working as unpaid family worker); unemployed; homemaker (busy with housework and/or family care); retired (retired, left work life due to age-related reasons)
What is your household's total net income per month?	Household Income - Categorized as lowest (2025 TL and below); low (2026-4052 TL); middle (4053-6890 TL); upper (6891 TL and above)
**Healthcare Service Accessibility**	
Was there any time in the past 12 months when you needed medical care but could not afford it? Was there any time in the past 12 months when you needed dental care but could not afford it? Was there any time in the past 12 months when you needed prescribed medicines but could not afford it? Was there any time in the past 12 months when you needed mental health care (e.g., by a psychologist or psychiatrist) but could not afford it?	Cost-related Unmet Need - The self-perceived cost-related unmet need for any healthcare service was defined as any positive answer to these four questions
Have you experienced delay in getting healthcare in the past 12 months because the time needed to obtain an appointment was too long?	Delay Due to Lengthy Appointment Scheduling (Previous Year) - Categorized as yes and no
Have you experienced delay in getting healthcare in the past 12 months due to distance or transport problems?	Delay Due to Distance or Transportation Constraints (Previous Year) - Categorized as yes and no
